# Replication-transcription complex of coronaviruses: functions of individual viral non-structural subunits, properties and architecture of their complexes

**DOI:** 10.18699/VJGB-22-15

**Published:** 2022-03

**Authors:** E.L. Mishchenko, V.A. V.A. Ivanisenko

**Affiliations:** Institute of Cytology and Genetics of the Siberian Branch of the Russian Academy of Sciences, Novosibirsk, Russia; Institute of Cytology and Genetics of the Siberian Branch of the Russian Academy of Sciences, Novosibirsk, Russia

**Keywords:** non-structural proteins CoVs, subunits of replicase CoVs, replication-transcription complex of CoVs, architecture of non-structural protein complexes CoVs, неструктурные белки коронавирусов (CoVs), субъединицы репликазы CoVs, репликационно-транскрипционный комплекс CoVs, архитектура комплексов неструктурных белков CoVs

## Abstract

Coronaviruses (CoVs) belong to the subfamily Orthocoronavirinae of the family Coronaviridae. CoVs are enveloped (+) RNA viruses with unusually long genomes. Severe acute respiratory syndrome CoV (SARS-CoV), Middle East respiratory syndrome CoV (MERS-CoV), and the novel coronavirus (2019-nCoV, SARS-CoV-2) have been identif ied as causing global pandemics. Clinically tested vaccines are widely used to control rapidly spreading, acute, and often severe infections; however, effective drugs are still not available. The genomes of SARS-CoV-2 and SARS-CoV are approximately 80 % identical, while the genomes of SARS-CoV-2 and MERS-CoV are approximately 50 % identical. This indicates that there may be common mechanisms of coronavirus pathogenesis and, therefore, potential therapeutic targets for each virus may be the same. The enzymes and effector proteins that make up the replication-transcription complex (RTC) of coronaviruses are encoded by a large replicase gene. These enzymes and effector proteins represent promising targets for potential therapeutic drugs. The enzyme targets include papain- and 3C-like cysteine proteinases that process two large viral polyproteins, RNA-dependent RNA polymerase, RNA helicase, viral genome-modifying enzymes, and enzymes with 3’–5’ exoribonuclease or uridylate-specif ic endonuclease activity. Currently, there are many studies investigating the complex molecular mechanisms involved in the assembly and function of the RTC. This review will encompass current, modern studies on the properties and complexes of individual non-structural subunits of the RTC, the structures of individual coronavirus RTC subunits, domain organization and functions of subunits, protein-protein interactions, properties and architectures of subunit complexes, the effect of mutations, and the identif ication of mutations affecting the viability of the virus in cell culture. Key words: non-structural proteins CoVs; subunits of replicase CoVs; replication-transcription complex of CoVs; architecture of non-structural protein complexes CoVs.

## Abstract (ru)

Коронавирусы относятся к семейству Coronaviridae подсемейства Orthocoronavirinae и представляют собой оболочечные (+) РНК-вирусы с необычно длинным геномом. В настоящее время часто идентифицируются, вызывая продолжающуюся пандемию во всем мире, коронавирусы Severe Acute respiratory syndrome CoV (SARS-CoV), Middle East respiratory syndrome CoV (MERS-CoV) и новый коронавирус (2019-nCoV, SARS-CoV-2). Для сдерживания быстро распространяющейся, острой и часто тяжело протекающей инфекции широко применяют прошедшие клинические испытания вакцины, однако эффективных лекарств по-прежнему нет. Геномы SARS-CoV-2 и SARS-CoV идентичны на ~80 %, а SARS-CoV-2 и MERS-CoV – на ~50 %. Это свидетельствует об общих механизмах патогенеза коронавирусов и одних и тех же потенциальных терапевтических мишенях. Ферменты и эффекторные белки, входящие в состав репликационно-транскрипционного комплек-са (РТК) коронавирусов, кодируются весьма крупным геном репликазы и представляют собой перспективные мишени действия потенциальных эффективных лекарств. Эти мишени включают папаин- и 3С-подобные цистеиновые протеиназы, осуществляющие процессинг двух больших вирусных полипротеинов, РНК-зависимую РНК-полимеразу, РНК-хеликазу, ферменты, модифицирующие вирусный геном, ферменты, обладающие 3’–5’-экзорибонуклеазной и уридилат-специфичной эндонуклеазной активностью, а также важные эффекторные белки. В настоящее время изучение сложных молекулярных механизмов сборки и функционирования РТК находится на пике изучения. Обзор посвящен актуальным и современным исследованиям свойств индивидуальных неструктурных субъединиц РТК и их комплексов и включает изучение структур индивидуальных субъединиц РТК коронавирусов, доменной организации субъединиц и их функций, белок-белковые взаимодействий, свойств и архитектуры комплексов субъединиц, влияния мутаций, а также выявления мутаций, влияющих на жизнеспособность вируса в клеточной культуре.

## Introduction

The 2019 coronavirus infection has spread globally, often causing severe respiratory, intestinal, and systemic illnesses. Coronaviruses (CoVs) belong to the Orthocoronavirinae subfamily of the Coronaviridae family. The subfamily is further divided into α-, β-, ϒ-, and δ-coronaviruses. Severe acute respiratory syndrome CoV (SARS-CoV), Middle East respiratory syndrome CoV (MERS-CoV), novel coronavirus (2019-nCoV, SARS-CoV-2), mouse hepatitis virus (MHV), and bovine coronavirus (BCoV) are all β-coronaviruses (Malik et al., 2020). Coronaviruses are enveloped viruses with an unusually long, single-stranded (+) RNA genome (26–32 kb). The SARS-CoV-2 genome is similar to the SARS-CoV genome (sequence identity ~80 %), while the SARS-CoV-2 and MERS-CoV genomes are less similar (sequence identity ~50 %) (Lu et al., 2020). The structure and function of proteins are preserved at levels as low as 30 % of amino acid sequence identity (Rost, 1999). This indicates that there may be common mechanisms of pathogenesis among the CoVs and, therefore, the viruses may have the same potential therapeutic targets. The 5′ proximal region of each CoV genome includes a cap, a 5′ untranslated region (UTR) and a long replicase gene encoding 16 non-structural proteins (comprising two-thirds of the genome). The 3′ regions encode structural proteins, including S (spike), E (surface), M (membrane), and N (nucleocapsid), auxiliary proteins (the number of these varies among CoVs), a 3′ UTR, and a poly(A) tract.

The replication-transcription complex (RTC) of CoVs is a complex consisting of viral and, probably, cellular proteins. The RTC produces the (+) RNA genome and a set of subgenomic CoVs RNA in infected cells. The CoV replicase gene has two overlapping open reading frames, ORF1a and ORF1b, which encode the viral components of the RTC. Expression of the gene leads to the formation of the pp1a polyprotein, which is encoded by ORF1a. A ribosomal frameshift of –1 before the ORF1a translation termination codon and ORF1b are required for the formation of the pp1ab polyprotein, which is a continuation of pp1a. Polyproteins pp1a and pp1ab are processed by two viral cysteine proteinases, papain-like proteinase PLpro (PLP) and 3C-like proteinase 3СLpro (Mpro), which results in the release of intermediate precursors and 16 mature highly conserved non-structural proteins (nsps) capable of associating with each other and being subunits of the RTC. The pp1a polyprotein includes nsp1–nsp11, while pp1ab includes all pp1a nsps, as well as nsp12–nsp16 (Nagvi et al., 2020).

## Structural and functional properties of conserved non-structural RTC subunits and their complexes

The molecular mechanisms of the assembly and function of the RTC has not been studied. However, the structural and functional properties of conserved non-structural RTC subunits and their complexes have been extensively researched and are extremely important for the identification of key drug targets against CoVs: nsp1 interacts with the 40S ribosome subunit and inhibits translation initiation of host proteins, including interferon response factors. Interaction of nsp1 with ribosomes also leads to the degradation of host RNA. Thus, nsp1 suppresses cellular defence antiviral mechanisms (Kamitani et al., 2006; Narayanan et al., 2008);

nsp2 is not part of the RTC in cell culture. However, the absence of nsp2 in cells infected with the MHVΔnsp2 or SARS-CoVΔnsp2 deletion mutants reduces the production of the virus and viral RNA (Graham et al., 2005);

nsp3 and nsp5 are proteinases that process the pp1a and pp1ab polyproteins, resulting in the release of individual RTC components. The PLP nsp3 domain(s) process the N-proximal regions of pp1a and pp1ab. The MHV nsp3 has two domains, PL1pro (PL1P) and PL2pro (PL2P). The PL1P domain cleaves the nsp1/nsp2 and nsp2/nsp3 sites, while the PL2P domain cleaves the nsp3/nsp4 site (Hughes et al., 1995; Kanjanahaluethai et al., 2000). The SARS-CoV nsp3 has a single PL2P domain that cleaves all three nsp sites (Thiel et al., 2003). The SARS-CoV PLP is an intracellular immune response antagonist. PLP blocks the activation of transcription factors IRF3 and NF-κB, which induces the expression of IFN(I) and antiviral genes. It does this by indirectly inhibiting IKKi and TBK1 kinases that activate IRF3 and stabilizing IκBα, an inhibitor of NF-κB (Frieman et al., 2009). PLP also hydrolyzes elements of ubiquitin and the product of interferon-stimulating gene 15 of the ubiquitin-like protein, thereby blocking the cellular mechanism of post-translational ubiquitination and, in turn, enhancing viral replication (Daczkowski et al., 2017). However, nsp3 stabilizes the host E3 ubiquitin ligase RCHY1 through the interaction of its SUD and PLP domains with RCHY1. This activates the RCHY1-mediated degradation of p53, a cellular inhibitor of SARS-CoV replication (Ma- Lauer et al., 2016). SARS-CoV nsp3 interacts with nsp5, nsp6, nsp12, nsp13, nsp14, and nsp16 in the yeast two-hybrid (Y2H) system and is thought to serve as a scaffold for RTC assembly (Imbert et al., 2008);

nsp5 is a 3CLpro (Mpro). Mpro plays a key role in the processing of pp1a and pp1ab polyproteins, cleaving the central and C-proximal regions of pp1ab at 11 highly conserved sites, which releases mature nsp4–nsp16 proteins (Ziebuhr et al., 2000; Thiel et al., 2003; Goyal B., Goyal D., 2020). Mpro is only active as a dimer. Self-elimination of MERS-CoV Mpro at the nsp4/nsp5 and nsp5/nsp6 sites occurs as a result of the ligand-induced formation of an “immature dimer” during the convergence of Mpro III domains within two polyproteins (Tomar et al., 2015). Structural analysis of the SARS-CoV-2 Mpro complexes with known antiviral inhibitors, Boceprevir (peptidomimetic NS3/4A protease of hepatitis C virus) and GC376 (inhibitor of CoV replication), revealed atomic-level interactions between Mpro and these inhibitors. Such studies are important for the optimization and design of effective drugs against CoVs (Fu et al., 2020);

nsp6 interacts with nsp2, nsp8, and nsp9 in the Y2H system (Brunn et al., 2007). Six of the predicted hydrophobic domains of MHV nsp6 and SARS-CoV nsp6 are transmembrane domains (Oostra et al., 2008). MHV nsp6 and SARS-CoV nsp6 are localized in the membranes of the endoplasmic reticulum (ER) and induce the formation of autophagosomes from ER membranes and activate autophagy (Cottam et al., 2011). Co-transfection of nsp3, nsp4, and nsp6 induces a change in the internal membranes of the host cell through the formation of double-membrane vesicles (DMVs), similar to the DMVs induced by SARS-CoV (Angelini et al., 2013);

and nsp8 co-crystallize to form the nsp7/8 hexadecameric supercomplex. The assembly of the supercomplex involves the formation of two different nsp7/8 heterodimers, D1 and D2, which differ in nsp8 conformation. D1 and D2 each dimerize to form the heterotetramers T1 and T2. The interaction of two T1 with two T2, in the order T1–T2–T1′–T2′ and with ring closure through the T1–T2′ interaction, leads to the construction of the full supercomplex. The supercomplex has a unique architecture: 16 molecules (8 nsp7 molecules and 8 nsp8 molecules) interact tightly with each other, forming a hollow cylindrical structure in which two nsp8 conformations coexist. The positive charge of the inner channel of the cylinder and its diameter (30 Å) indicates the ability of the nsp7/8 supercomplex to surround and interact with doublestranded RNA (dsRNA) (Zhai et al., 2005). The SARS-CoV nsp7/8 hexadecameric supercomplex can be formed in solution at an equimolar nsp7: nsp8 ratio (Zhai et al., 2005; Velthuis et al., 2012).

In solution, hexadecameric SARS-CoV nsp7/8 associates with dsRNA (Kd ~1.2 μM). The association of nsp7/8 with dsRNA is mediated by nsp8 and enhanced by nsp7 (Velthuis et al., 2012). On its own, SARS-CoV nsp8 possesses primerindependent RNA-dependent RNA polymerase (RdRp) activity and initiates, with low fidelity, the de novo synthesis of short (less than 6 nucleotides) complementary oligomers (primers) on single-stranded RNA (ssRNA) templates (Imbert et al., 2006). SARS-CoV nsp8 and nsp7/8 complex also exhibit primer-dependent RdRp activity (Velthuis et al., 2012). The FCoV (feline coronavirus) nsp7/8 complex is a 2 : 1 heterotrimer formed by the association of two nsp7 molecules and one nsp8 molecule. This complex does not form a hollow structure, either in crystalline form or in solution. FCoV nsp7/8 has primer-independent RdRp activity (Xiao et al., 2012);

nsp9 is an ssRNA-binding protein (Egloff et al., 2004; Miknis et al., 2009). Both monomeric and dimeric forms of PDCoV (porcine δ coronavirus) nsp9 and PEDV (porcine epidemic diarrhoea virus related to α coronaviruses) nsp9 (Zeng et al., 2018), as well as the dimeric form of SARSCoV nsp9 (Miknis et al., 2009), have been found in solution during in vitro experiments. Studies of the crystal structures of SARS-CoV nsp9 (Egloff et al., 2004; Miknis et al., 2009), PDCoV nsp9, and PEDV nsp9 (Zeng et al., 2018) have revealed dimeric forms of nsp9. The monomer SARS-CoV nsp9 is characterized by a different structure compared to other proteins involved in the replicative complexes of RNA viruses, the features of which are similar to the structures of oligosaccharide/oligonucleotide-binding proteins (Egloff et al., 2004). Mutations affecting the dimerization of SARS-CoV nsp9 weaken its interaction with ssRNA, which is lethal for SARS-CoV replication in cell culture (Miknis et al., 2009);

nsp10 interacts with dsRNA, dsDNA, and ssRNA with micromolar affinity (Joseph et al., 2006). Crystal structure studies of SARS-CoV nsp10 showed that the monomer structure includes two zinc fingers, a new discovery among zinc finger protein structures. Motifs of zinc-binding nsp10 sequences have been identified. Twelve identical monomers form a unique spherical dodecameric architecture, which is hypothesized to be the functional form of nsp10 (Joseph et al., 2006; Su et al., 2006). Through two-hybrid analysis in mammalian cells, interactions of SARS-CoV nsp10 with nsp14 and nsp16 have been revealed (Pan et al., 2008);

nsp11 is a short peptide resulting from the cleavage of the pp1a polyprotein by the 3CLpro/Mpro proteinase at the nsp10/ nsp11 site. Nsp11 is encoded by the region of genomic RNA where the translational reading frame shift occurs (ORF1a to ORF1b). This shift results in the formation of nsp12–nsp16 proteins from the pp1ab polyprotein. SARS-CoV-2 nsp11 contains 13 amino acid residues and has a disordered conformation, the dynamics of which have been studied in the presence of lipid-membrane mimetics. In the presence of SDS micelles, the disordered conformation of nsp11 is transformed into an α-helix (Gadhave et al., 2021);

nsp14 is bifunctional. The N-terminal domain of nsp14 has 3′–5′ exoribonuclease activity (ExoN), and its C-terminal domain has (guanine-N7) methyltransferase activity (N7-MTase). ExoN corrects the low fidelity of synthesis of the complementary RNA strand by viral RdRp nsp12 and catalyzes the removal of 3′-terminal erroneous nucleotides in dsRNA. N7-MTase catalyzes the methylation of the viral RNA cap at the N7 guanine position in the presence of S- adenosylmethionine (methyl group donor). N7-MTase has an S- adenosylmethionine binding motif which recognizes the cap of viral GpppRNA and methylates guanine N7 GpppRNA to form 7MeGpppRNA (cap-0). Cap-0 plays an important role in blocking the degradation of viral RNA by 5′–3′ exoribonucleases, translation initiation, and immune system control escape (Chen et al., 2009; Tahir, 2021). Mutants of the catalytic motif MERS-CoV ExoN and SARS-CoV-2 ExoN are not viable in cell culture (Ogando et al., 2020). SARS- CoV nsp10 associates with the ExoN domain of SARS-CoV nsp14, increasing the ExoN activity of nsp14 by more than 35 times without affecting its N7-MTase activity (Bouvet et al., 2012). Structural studies of the SARS-CoV nsp10/14 complex showed that one nsp10 molecule associates with the ExoN domain of nsp14, stabilizing and enhancing the activity of the ExoN. The architecture of the nsp10/14 complex has been studied and has been found to include two regions of contact between the nsp10 molecule and the ExoN domain of nsp14 (Ma et al., 2015);

nsp16 has (nucleoside-2′O) methyltransferase activity (2′O-MTase). 2′O-MTase recognizes the cap-0 of viral RNA and catalyzes the transfer of the methyl group from S-adenosylmethionine to the 2′OH group of the first nucleotide’s ribose after N7-methylated guanine, resulting in the formation of 7MeGpppN2′OMe-RNA (conversion of cap-0 into cap-1). SARS-CoV nsp10 associates with nsp16 and stimulates the 2′O-MTase activity of nsp16 (Bouvet et al., 2010). Mutagenesis mapping of the surface amino acid residues of SARS-CoV nsp10 involved in nsp10–nsp14 interaction and structural analysis of the nsp10/16 complex revealed overlapping surfaces of nsp10 interacting with nsp14 and nsp16. Nsp10 can serve as a platform that recruits nsp14 or nsp16 to the RTC, stimulating the ExoN activity of nsp14 or the 2′O- MTase activity of nsp16. Therefore, nsp10 is an important regulator of the RTC. Mutations have been identified that disrupt the nsp10–nsp14 and nsp10–nsp16 interactions, some of which lead to a nonviable viral phenotype in cell culture (Bouvet et al., 2014);

nsp12, an RdRp, catalyzes the synthesis of complementary RNA strands on (+) and (–) viral RNA templates and is a key CoV RTC enzyme. CoVs nsp12 initiates de novo synthesis from the 3′ end of the viral genome of the full-length (–) RNA strand, as well as for subgenomic (–) RNA transcripts, which have differing 3′ end lengths. In turn, the full-length (–) RNA strands and subgenomic (–) RNA strands serve as templates for the synthesis of the new RNA genome and subgenomic (+) RNA transcripts. Subgenomic (+) RNA is important for the expression of structural and accessory proteins encoded by genes in the 3′ proximal region of the viral genome, which is inaccessible to ribosomes that translate the viral genome (Pasternak et al., 2006). Full-length recombinant SARS-CoV nsp12 associates with short (20–30 nucleotides long) dsRNA and ssRNA (Kd 0.13 and 0.1 μM, respectively), and initiates primer-dependent RNA synthesis on both homo- and heteropolymeric RNA templates of the same length (Velthuis et al., 2010). However, the recombinant SARS-CoV nsp12 does not associate with a primer-template that mimics the 3′-terminal 40 nucleotides of the SARS-CoV UTR and does not exhibit RdRp activity on this primer-template (Subissi et al., 2014).

Cryo-electron microscopy determined the structure of the monomer SARS-CoV-2 nsp12, which includes the nidovirusspecific N-terminal domain of the RdRp-associated nucleotidyl transferase (NiRAN), the interface domain, and the C- terminal RdRp domain. The C-terminal RdRp domain has a conserved right-hand-like architecture, which includes three subdomains: fingers, palm, and thumb. The active centre is formed by conservative motifs of amino acid residues localized in the palm subdomain. The motifs of the channel of entry for nucleotide triphosphates and the primer-template and the exit of the resulting RNA strand converge in the central cavity, where these motifs carry out matrix-dependent RNA synthesis have been determined (Gao et al., 2020).

The association of the nsp7/8 complex with nsp12 leads to the formation of the nsp7/8/12 complex. This complex possesses high RNA-binding capacity, polymerase activity, and processivity. It is also capable of initiating de novo RNA synthesis on the 3′ UTR template of the SARS-CoV genome, resulting in the elongation of the RNA product by over 300 nucleotides. For nsp7/8/12 complex-mediated initiation of processive RNA synthesis, three amino acid residues from nsp7 (K7, H36, N37) and one amino acid residue from nsp8 (K58) are required for the interaction of nsp7/8/12 with the RNA template, while four amino acid residues from nsp8 (D99, P116, P183, R190) interact with nsp12 (Subissi et al., 2014). Moreover, nsp7/8/12 is able to associate with nsp14 to form the nsp7/8/12/14 multicomplex. This ensemble of non-structural proteins possesses high RNA polymerase activity and is involved in 5′ RNA capping; however, it does not have ExoN activity (Subissi et al., 2014). The structure of the SARS-CoV-2 and SARS-CoV nsp7/8/12 complexes was determined by cryo-electron microscopy. These complexes, including the nsp12 monomer, nsp8 monomer, and nsp7/nsp8 heterodimer, have similar architecture: the nsp8-1 subunit interacts with the RdRp finger subdomain, while the nsp7 and nsp8-2 subunits interact with the RdRp thumb subdomain (Gao et al., 2020);

nsp13 possesses helicase and nucleoside-triphosphatase (NTPase) activities; nsp13 interacts with nsp7, nsp8, and nsp12 in the Y2H system (Brunn et al., 2007; Pan et al., 2008). The crystal structure of MERS-CoV nsp13 was determined. The structure includes an N-terminal zinc-binding domain (ZBD) rich in Cys/His residues that coordinate three zinc ions, as well as C-terminal helicase RecA1 and RecA2 domains which contain parallel β-chains (Hao et al., 2017). The nsp13 helicase separates dsRNA and dsDNA strands with overhanging (5–20 nucleotides long) 5′ ends; nsp13 interacts with the single-stranded 5′ end of the partial nucleic acid duplex of dsRNA and dsDNA and unwinds it in the 5′–3′ direction, using the hydrolysis energy of the 5′-deoxy- and ribonucleotide triphosphates (Ivanov et al., 2004; Adedeji et al., 2012, 2016). The RNA 5′-triphosphatase activity of nsp13 catalyzes the cleavage of the ϒ phosphate from the 5′-terminal nucleotide of RNA and is thought to be involved in the capping of viral RNA (Ivanov et al., 2004). Studies of SARS-CoV nsp13 have shown that the unwinding of DNA duplexes occurs in discrete intervals of ~9.3 base pairs (bp) at a rate of 30 intervals per second. Therefore, the unwinding speed of DNA duplexes is approximately 280 bp/s. The helicase activity of nsp13 increases approximately 2-fold when nsp13 interacts with nsp12, suggesting the interaction of these proteins is involved in the function of the RTC (Adedeji et al., 2012). When SARS-CoV-2 nsp13 interacts with the RTC, specifically with nsp7/2nsp8/nsp12: RNA, the stable complexes 2nsp13–RTC (67 %) and nsp13– RTC (20 %), as well as the dimer (2nsp13–RTC)2 (13 %) are formed. Using cryo-electron microscopy, the architecture of the dominant 2nsp13–RTC complex has been determined. This involves the interaction of the ZBD on the first nsp13 molecule with the N-terminus of nsp8b and the nsp12 thumb subdomain, as well as the interaction of the ZBD of the second nsp13 molecule with the N-terminus of nsp8a. The catalytic RecA1 helicase domain of the first nsp13 molecule is fixed against nsp7 and the nsp8b head (Chen et al., 2020);

nsp15 is a nidovirus uridylate-specific endoribonuclease (NendoU). It cleaves RNA at the 3′ uridylate in unpaired, single-stranded, and looped regions (Bhardwaj et al., 2006; Zang et al., 2018). The crystal structures of SARS-CoV-2, SARS-CoV, and MERS-CoV nsp15 are homologous, functionally active hexamers formed by the dimerization of trimers. Each of the hexamer protomers includes three domains: the N- terminal, middle, and C-terminal catalytic NendoU domains. During trimer assembly, the N-terminal domain of one protomer is packed into a gap between the central and C-terminal domains of the neighbouring protomer. During assembly of the hexamer, the N-terminal domains of the protomers of the two trimers are packed back-to-back and are in the centre of the hexamer structure. The C-terminal domains containing active centres are located outward at the vertices of the cloverleaf. This architecture provides nsp15 with six functionally active centres (Zang et al., 2018; Kim et al., 2020).

The uridylate content in RNA, Mn+2 and, to a lesser extent, Mg+2 increase the affinity of nsp15 for RNA. The hexameric structure of SARS-CoV and MERS-CoV nsp15 is critical for its substrate and catalytic activity. The study of a series of mono-, tri- and hexameric protein mutants revealed weak interactions with RNA (rU16–20) and low catalytic activity in monomers and trimers compared to hexamers and wild-type nsp15 (Bhardwaj et al., 2006; Zang et al., 2018). Nsp8 and the nsp7/8 complex interact with MERS-CoV nsp15, enhancing the binding ability of the nsp15 hexamer for RNA and its catalytic activity (Zang et al., 2018). The NendoU activity of nsp15 is an antagonist of the IFN-induced cellular antiviral response and stimulates the initiation of viral RNA translation (Deng et al., 2018, 2019).

A large number of non-structural subunits and their respective complexes within the RTC is a defining feature of CoVs. An important feature is that some subunits have domains with different enzymatic activities. For example, nsp14 has both ExoN and N7-MTase activity, while nsp13 has helicase and nucleoside triphosphatase activity. Non-structural proteins have a set of activities universal for (+) RNA viruses: proteinases (nsp3, nsp5), RNA-dependent RNA polymerases (nsp12), RNA helicases (nsp13). There are also unique domains involved in mRNA capping, cap modification (nsp14, nsp16). Some proteins possess 3′–5′ exoribonuclease activity (nsp14), which regulate the reliability of RNA genome replication, while some possess uridylate-specific endonuclease activity (nsp15). Many proteins serve as cofactors for important enzymes (nsp7, nsp 8, nsp10) and affect cellular processes. In particular, some proteins suppress the antiviral cellular response (nsp1, nsp3, nsp6, nsp15). The nsp9 and nsp10 structures are unique among the protein structures of the replicative complexes of RNA viruses. Many CoVs subunits and their complexes have a complex architecture. For example, nsp7 and nsp8 form a functional and unique hexadecameric supercomplex, the nsp10 architecture includes 12 identical subunits, and the nsp15 architecture is a functionally active hexamer. The complex architecture of the RTC is defined by the 2 nsp13/2 nsp8/1 nsp7/1 nsp12 model.

## Conclusion

The lack of effective drugs against the novel coronavirus infection is a current global challenge. The RTC of CoVs replicates (+) RNA and determines the production of the virus in infected cells; however, the molecular mechanisms of this remain unexplored. Currently, great efforts are being undertaken aimed at creating a structural or functional network of interactions within the CoV proteome, as well as its interactions with the host cell. This would identify a large-scale panel of therapeutic targets. The determination of the structures of individual non-structural RTC subunits and their complexes and the identification of key interacting amino acid residues and types of bonds between them will enable the design of selective and effective inhibitors. Investigations employing biochemical methods and mutational analyses can identify factors that affect the efficiency of viral genomic RNA production and the virus in an infected cell, the multiple effects of viral proteins on the host cell, and potential key drug targets.

## Conflict of interest

The authors declare no conflict of interest.
